# Comparison of survival outcomes of neoadjuvant therapy and direct surgery in IB2/IIA2 cervical adenocarcinoma: a retrospective study

**DOI:** 10.1007/s00404-020-05505-6

**Published:** 2020-03-27

**Authors:** Peilin Ouyang, Jingting Cai, Lin Gui, Shan Liu, Na-Yi Yuan Wu, Jing Wang

**Affiliations:** 1 Hunan Cancer Hospital/The Affiliated Cancer Hospital of Xiangya School of Medicine, Central South University, 283, Tongzipo Road, Changsha, Hunan People’s Republic of China; 2 Xiangya School of Medicine, Central South University, Changsha, Hunan People’s Republic of China

**Keywords:** Cervical adenocarcinoma, NACT, NACRT, Radical surgery, Survival

## Abstract

**Purpose:**

This retrospective study compared the efficacy and survival of patients with cervical adenocarcinoma (IB2/IIA2; FIGO2009) treated with neoadjuvant chemotherapy before radical surgery (NACT + RS), neoadjuvant chemoradiation therapy before radical surgery (NACRT + RS), or primary radical surgery (RS).

**Methods:**

Between January 2008 and November 2015, 91 patients diagnosed with stage IB2/IIA2 cervical adenocarcinoma were enrolled, including 29 patients who received RS, 24 patients who received NACT + RS, and 38 patients who received NACRT + RS.

**Results:**

The characteristics of patients were balanced among the three groups, and the median follow-up time was 72 months. The 5 year disease-free survival (DFS) rate was 75.8% and the 5 year overall survival (OS) rate was 85.0%. Univariate analysis revealed that effectiveness of neoadjuvant treatment, tumor size, lymph node metastases, and depth of stromal invasion were the factors predicting recurrence and mortality. Multivariate Cox proportional analysis revealed that the occurrence of a lymph node metastasis was an independent prognostic factor of DFS (hazard ratio [HR] = 0.223; 95% confidence interval [CI]: 0.060–0.827) and OS (HR = 0.088; 95% CI: 0.017–0.470). On survival analysis of preoperative adjuvant chemotherapy and primary surgery, the 5 year OS (*P* = 0.010) and DFS (*P* = 0.016) rates for the NACRT + RS group were significantly lower than those for the RS group.

**Conclusion:**

Stage IB2/IIA2 cervical adenocarcinoma patients who received primary RS had a better DFS and OS than those who received preoperative NACRT. There was no significant difference when compared to the preoperative NACT group.

## Introduction

There were an estimated 570,000 cervical cancers diagnosed and 311,000 deaths from cervical cancer worldwide in 2018 [1]. The most common cervical histology is squamous cell carcinoma (SCC); cervical adenocarcinoma (AC) accounts for approximately 15–20% of cervical cancer cases. AC is considered to develop within the cervix and has no obvious symptoms in the early stages; therefore, it is usually diagnosed in advanced stage [2]. AC incidence rates have increased in Europe, and Finland, Slovakia, and Slovenia by approximately 3% in recent decades [3].Similarly, the proportional incidence of cervical AC has also increased in the United States [4]. A population-based analysis demonstrated that the 5 year survival rates for stage IB2 and IIA SCC were 69 and 58.3%, respectively, whereas those for stage IB2 and IIA AC were 68.3 and 45.5%, respectively, obviously lower than those for SCC [5]. Concurrent chemoradiotherapy (CCRT) is regarded as the primary treatment for locally advanced cervical cancer (LACC) based on benefits of lower regional recurrence and longer survival outcomes compared to radical surgery (RS) [6]. Several studies have pointed out that regardless of treatment (i.e., definitive radiotherapy (RT) or CCRT), the survival benefit for AC is lower than that for SCC [7, 8]. There are several potential reasons for this observation: (1) survival outcomes of AC may affected by different subtypes, such as gastric type AC, clear cell AC, and endometrial endometrioid AC; (2) AC is not as sensitive to RT as SCC [7, 9, 10], which also results in high rates of locoregional failure [8]; and (3) compared to SCC, AC’s biological characteristics are more radical, including higher local recurrence, lymph node metastasis, and distant metastasis rates than SCC [11]. Therefore, it is important to distinguish AC from SCC and formulate more individualized treatment protocols. Studies have shown that preoperative neoadjuvant chemotherapy (NACT) is well tolerated and beneficial in reducing tumor size, can improve long-term disease-free survival (DFS) and overall survival (OS), and the accuracy of frozen section examination in pelvic lymph node operation is not affected [9, 12]. Although multiple studies have found that NACT before RS does not improve OS in patients with local AC when compared to CCRT or RS alone [13–17], whether cervical AC benefits from neoadjuvant therapy is still uncertain. The purpose of this retrospective study was to compare the outcomes of platinum-based NACT followed by radical hysterectomy and radical hysterectomy alone for patients with cervical AC.

## Materials and methods

### Patients

This retrospective study included 91 patients with histologically diagnosed cervical IB2/IIA2 AC who underwent radical hysterectomy with pelvic lymphadenectomy between January 2008 and November 2015 at the Division of Gynecologic Oncology at the affiliated Cancer Hospital of Xiangya School of Medicine. Pre-treatment evaluation included medical history, European Cooperative Oncology Group (ECOG) performance status, gynecologic and physical examination, laboratory exams, tumor biopsy, and chest-abdominal–pelvic computed tomography. Patient information was taken from the hospital’s case recording system, including the treatment process (NACT regimen, pathological characteristics, and surgical records).

### Eligibility

Inclusion criteria were as follows: (1) adenocarcinoma histologically confirmed by two pathologists based on the World Health Organization staging system for tumors of the uterine cervix; (2) FIGO stages IB2 and IIA2 as determined by two or more gynecologic oncologists. Clinical staging was performed according to the International Federation of Gynecology and Obstetrics staging criteria (FIGO2009); (3) no concomitant malignancy or prior invasive malignancy; (4) at least two cycles of chemotherapy in patients who received NACT; (5) no other serious complications before treatment; and (6) ECOG performance status ≥ 2. Patients with histologically confirmed SCC, adenosquamous carcinoma, neuroendocrine carcinoma, clear cell carcinoma, and other scarce histologies were excluded from this study.

### Ethics approval and consent to participate

The project was licensed by the Hunan Cancer Hospital ethics committee (project number: 2015[01]) and the Chinese Clinical Trial Registry (registration number: ChiCTR1800018931).

### Treatments

All patients underwent radical hysterectomy type III (Piver-Rutledge classification) [18]. 11 patients received laparoscopy, 80 received laparotomy, and only one patient underwent adnexal preservation. 29 patients received RS directly (RS group) and 24 patients received 2–3 cycles of platinum-based NACT before surgery (NACT + RS group). The following chemotherapy regimens were administered: TP: paclitaxel 135–175 mg/m^2^ on day 1 and cisplatin 50 mg/m^2^ on day 2, repeated every 3 weeks; TC: paclitaxel 135–175 mg/m^2^ on day 1 and carboplatin area under the curve 5 mg/mL/min on day 2, repeated every 3 weeks; and TN: paclitaxel 135–175 mg/m^2^ on day 1 and nedaplatin 75–80 mg/m^2^ on day 2, repeated every 3 weeks. 38 patients underwent 3–4 cycles of brachytherapy followed by one cycle of platinum-based NACT (NACRT + RS group). ^192^Ir intracavity brachytherapy was delivered at a dose of 600 cGy at point A twice a week. The indication for adjuvant radiation therapy was the following risk factors: lymph node involvement, compromised surgical margin, parametrial infiltration, and depth of stromal invasion more than half of the cervix. Patients with risk factors received external beam radiation therapy (EBRT) and brachytherapy with concomitant chemotherapy. There were 36 patients who underwent postoperative EBRT because of lymph node involvement or deep stromal invasion: 11 in the NACT + RS group, 13 in the NACRT + RS group, and 12 in the RS group. No patients underwent EBRT because of parametrial infiltration or compromised surgical margin. Patients received postoperative EBRT with total dose of 45 Gy in 23–25 fractions, followed by brachytherapy twice a week for each A point at 600 cGy for a total dose of 42 Gy. During radiotherapy, patients received platinum-based chemotherapy on a 1 week cycle.

### Evaluation of short-term response

Complete response (CR) was defined as complete disappearance of the cervical lesions without lymph node metastasis. Optimal partial response (OPR) was defined as residual lesion interstitial infiltration less than 3 mm with or without lymph node metastasis. Pathological optimal response (pOR) was the total number of cases with CR and OPR. Pathological suboptimal response included persistent residual disease with more than 3 mm interstitial infiltration in the surgical specimen. The pathological evaluation was performed by two pathologists.

### Statistical analysis

OS was defined as the time from the start of the study to the date of death or the last date the patient was seen. DFS was defined as the date of surgery to the date of recurrence. The date of death was confirmed by the local government or hospital follow-up records. OS and DFS curves were calculated by the Kaplan–Meier method, and statistical differences between each group were evaluated by the log-rank test. Cox proportional hazard models were performed to estimate hazard ratios (HRs). *P* values less than 0.05 were considered significant. SPSS version 22.0 was used for statistical analysis.

## Results

### Patient characteristics

**Fig. 1:**
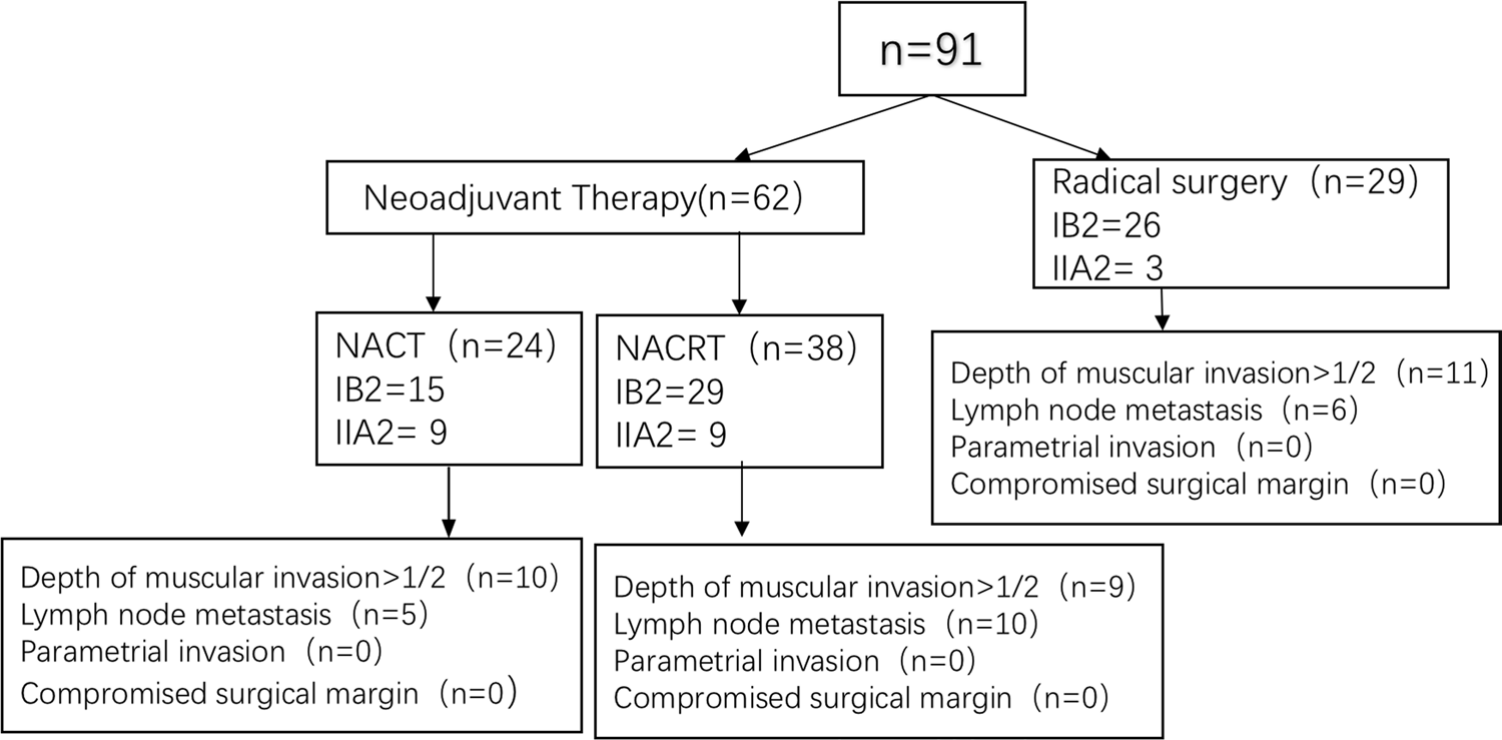
The group of experiment and the process of treatment

From January 2008 to November 2015, a total of 91 patients diagnosed with stage IB2/IIA2 cervical AC were eligible for this study (Fig. 1). The patient characteristics are displayed in Table 1. The mean age was 47 years (range 22–65 years). There were 24 patients in the NACT + RS group, 38 patients in the NACRT + RS group, and 29 patients in the RS group. 21 patients had lymph node metastasis: 5 patients in the NACT + RS group, 10 patients in the NACRT + RS group, and 6 patients in the RS group. 30 patients had deep stromal invasion: 10 patients in the NACT + RS group, 9 patients in the NACRT + RS group, and 11 patients in the RS group.

**Table 1:** Patients characteristics

Item	NACT + RS (*n* = 24) *N *(%)	NACRT + RS (*n* = 38) *N *(%)	RS (*n* = 29) *N *(%)	*P*
Age (years)				
< 47	17 (70.8%)	25 (65.8%)	19 (65.5%)	0.899
≥ 47	7 (29.2%)	13 (34.2%)	10 (34.5%)	
Tumor size (cm)				
≤ 5	10 (41.7%)	15 (39.5%)	17 (58.6%)	0.458
> 5	14 (58.3%)	23 (60.5%)	12 (41.4%)	
Histological grade				
Grade 1	9 (37.5%)	19 (50.0%)	8 (27.6%)	0.413
Grade 2	11 (45.8%)	16 (42.1%)	16 (55.2%)	
Grade 3	4 (16.7%)	3 (7.9%)	5 (17.2%)	
Lymphnode metastasis				
Positive	5 (20.8%)	10 (26.3%)	6 (20.7%)	0.825
Negative	19 (79.2%)	28 (73.7%)	23 (79.3%)	
Stage				
IB2	15 (62.5%)	29 (76.3%)	26 (89.7%)	0.062
IIA2	9 (37.5%)	9 (23.7%)	3 (10.3%)	
Depth of muscular invasion				
< 1/2	14 (58.3%)	29 (76.3%)	18 (62.1%)	0.269
≥ 1/2	10 (41.7%)	9 (23.7%)	11 (37.9%)	

*NACT* neoadjuvant chemotherapy, *NACRT* neoadjuvant chemo-radiation therapy, *RS* radical surgery

### Effectiveness and toxicity of neoadjuvant therapy

Thirty patients who underwent neoadjuvant therapy achieved pOR: 10 in the NACT group (41.7%) and 20 in the NACRT group (52.6%) (Table 2). The incidences of myelosuppression, hepatic and renal dysfunction, and sensory neuropathy were similar between the two groups. The incidence of nausea/vomiting in the NACT group was higher than that in the NACRT group. 1 patient in the NACT group developed grade 3 neutropenia with TP and 2 patients in the NACRT group developed grade 3 neutropenia with TC and TP, respectively. All of these patients had normal neutrophil levels after administration of granulocyte colony-stimulating factor. No patients discontinued neoadjuvant therapy due to therapeutic toxicity (Table 3). The cumulative toxicities of postoperative pelvic EBRT are listed in Table 4. Adverse events of postoperative pelvic EBRT included one case of proctitis in the RS group, two cases of vaginal stenosis and one case of cystitis in the NACRT + RS group, and one case of dermatitis and one case of vaginal stenosis in the NACT group.

**Table 2:** Effectiveness of neoadjuvant therapy

Terms	CR (*n*)	OPR (*n*)	SR (*n*)
NACRT			
B2	6	9	14
IIA2	1	4	4
NACT			
IB2	1	6	8
IIA2	0	3	6

*NACT* neoadjuvant chemotherapy, *NACRT* neoadjuvant chemo-radiation therapy, *CR* complete response, *OPR* optimal partial response, *SR* suboptimal response

**Table 3:** Summary of Grade ≥ 3 adverse events

Adverse event	NACT (*n *= 24)			NACRT (*n *= 38)		
	TC	TP	TN	TC	TP	TN
	5	6	13	8	18	12
Grade 3–4 myelosuppression	0	1	0	1	1	0
Grade 3–4 creatinine	0	0	1	1	1	0
Grade 3–4 hypohepatia	1	1	1	1	1	0
Nausea/vomiting	2	3	2	1	2	0
Sensory neuropathy	1	1	1	1	1	1

*NACT* neoadjuvant chemotherapy, *NACRT* neoadjuvant chemo-radiation therapy, *TC* paclitaxel plus carboplatin, *TP* paclitaxel plus cisplatin, *TN* paclitaxel plus nedaplatin

**Table 4:** Cumulative toxicity of postoperative pelvic EBRT

Site	NACT (*n* = 11)	NACRT (*n* = 13)	RS (*n* = 12)
Proctitis	0	0	1
Cystitis	0	1	0
Vaginal stenosis	1	2	0
Dermatitis	1	0	0

*NACT* neoadjuvant chemotherapy, *NACRT* neoadjuvant chemo-radiation therapy, *RS* radical surgery

### Survival

**Fig. 2:**
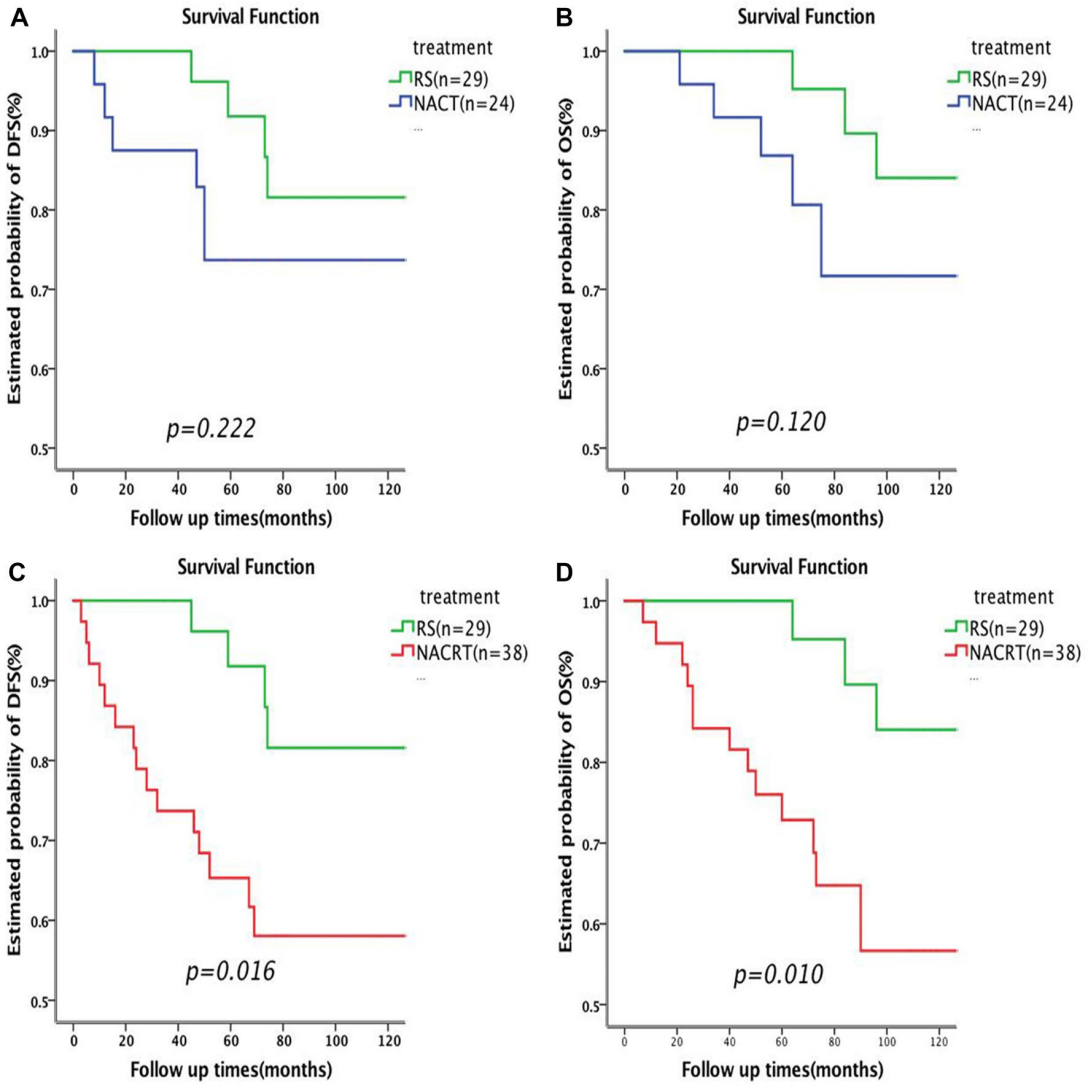
Plot of Kaplan–Meier disease free survival (**a**) and overall survival (**b**) for the NACT + RS group compared with RS alone group and for disease free survival (**c**) and overall survival (**d**) for the NACRT + RS group compared with RS alone group. *NACT* neoadjuvant chemotherapy, *RS* radical surgery, *NACRT* neoadjuvant chemoradiation therapy

**Fig. 3:**
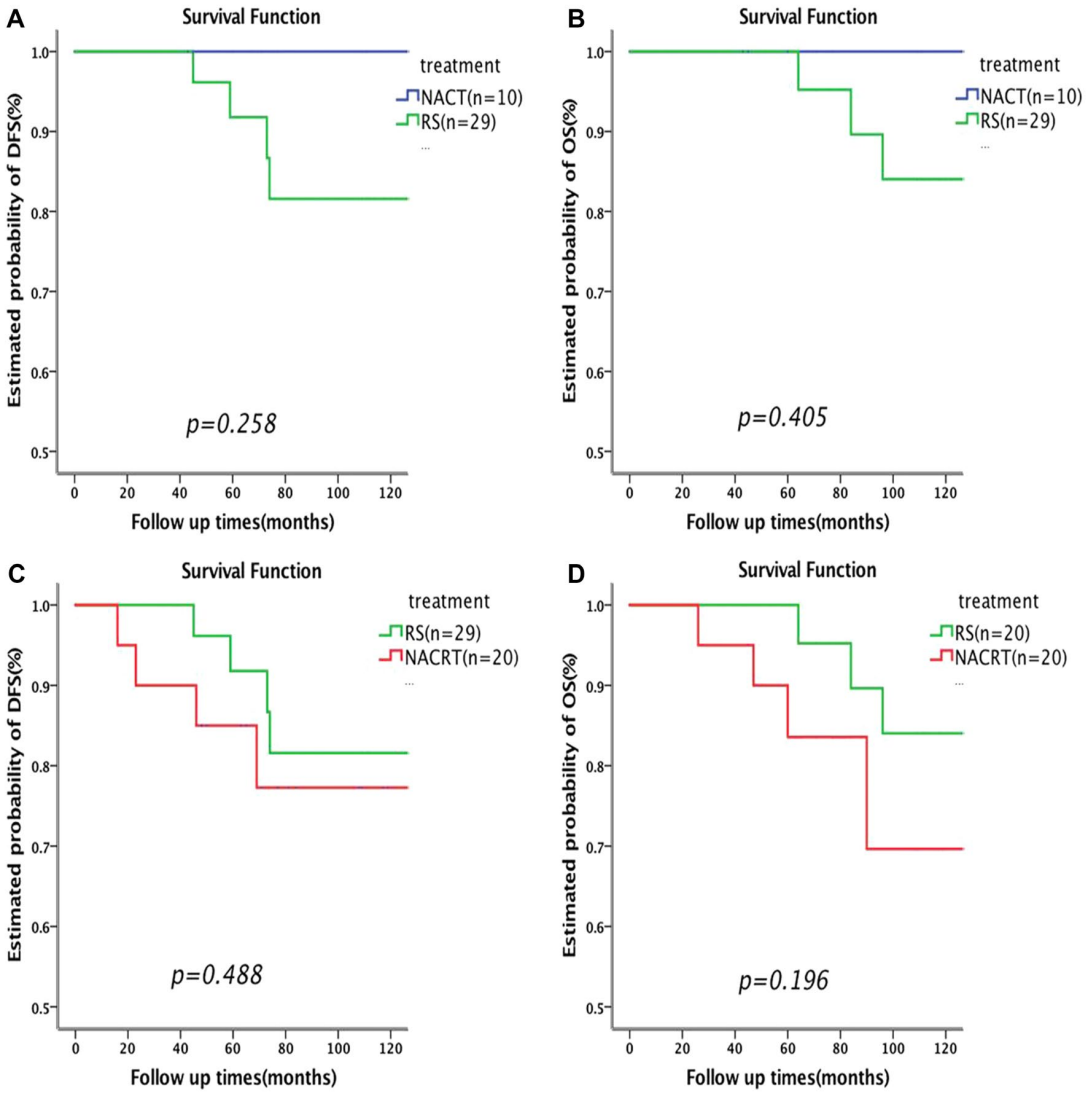
Plot of Kaplan–Meier estimates for disease free survival (**a**) and overall survival (**b**) for the patients with optimal response in the NACT + RS group compared to the RS group and for disease free survival (**c**) and overall survival (**d**) for the patients with optimal response in the NACRT + RS group compared to the RS group. *NACT* neoadjuvant chemotherapy, *RS* radical surgery, *NACRT* neoadjuvant chemoradiation therapy

The 5 year DFS rates for patients undergoing NACT + RS, NACRT + RS, and RS were 73.7, 68.4, and 91.8%, respectively (*P* = 0.053). The 5 year OS rates were 86.8% for patients receiving NACT + RS, 72.9% for those receiving NACRT + RS, and 100.0% for those undergoing RS (*P* = 0.035). There were 21 patients with positive lymph nodes who were treated with postoperative CCRT. The 5 year DFS rate in this group was 37.0%, and the 5 year OS rate was 60.8% (Table 5). DFS and OS curves by treatment group are shown in Fig. 2. There was no statistically significant difference between the RS group and the NACT + RS group in the 5 year DFS (91.8% vs 73.7%, *P* = 0.222, Fig. 2a) or the 5 year OS (100.0% vs 82.9%, *P* = 0.120, Fig. 2b). However, the 5 year DFS in the RS group was significantly higher than that in the NACRT + RS group (91.8% vs 65.3%, *P* = 0.016, Fig. 2c); the same was observed for the 5 year OS (100.0% vs 72.9%, *P* = 0.010, Fig. 2d). DFS and OS curves of the patients with pathological optimal response in the neoadjuvant therapy group compared to the RS group are shown in Fig. 3.

**Table 5:** Univariate analysis of disease-free survival and overall survival

Variable	Pts	5 year DFS (%)	*P* value	5 year OS (%)	*P* value
Tumor size (cm)					
≤ 4	42	83.3	0.019	90.5%	0.003
> 4	49	69.1		80.0%	
Lymphnode metastasis					
Negative	70	88.0	0.000	92.7%	0.000
Positive	21	37.0		60.8%	
Stage					
IB2	70	80.2	0.082	91.0%	0.010
IIA2	21	61.2		64.0%	
Depth of muscular invasion					
< 1/2	61	81.1	0.005	87.9%	0.018
≥ 1/2	30	65.4		79.4%	
Effectiveness of neoadjuvant therapy					
OR	30	89.7	0.001	68.5%	0.007
SR	32	49.8		88.9%	
Treatment					
NACT	24	73.7	0.053	86.8%	0.035
NACRT	38	68.4		72.9%	
RS	29	91.8%		100%	

*NACT* neoadjuvant chemotherapy, *NACRT* neoadjuvant chemo-radiation therapy, *RS* radical surgery, *DFS* disease free survival, *OS* overall survival, *OR* optimal response, *SR* suboptimal response

### Risk factors for recurrence and death

The results of the univariate analyses are shown in Table 5. Tumor size, lymph node metastasis, treatment type, and response to neoadjuvant therapy were significant variables for both DFS and OS. Multivariate analysis by Cox proportional hazards model showed that lymph node metastasis was an independent prognostic factor for DFS and OS (Table 6).

**Table 6:** Multivariate analysis of recurrence-free survival and overall survival

Varient	DFS		OS	
	HR (95% CI)	*P*	HR (95% CI)	*P*
Age (years)	1.901 (0.465–7.774)	0.371	1.881 (0.327–10.820)	0.479
Tumor size (cm)	0.915 (0.242–3.452)	0.895	0.459 (0.104–2.026)	0.304
Histological grade	NA	0.135	NA	0.134
Lymphnode metastasis	0.223 (0.060–0.827)	0.025	0.088 (0.017–0.470)	0.004
Stage	0.621 (0.149–2.590)	0.513	0.401 (0.073–2.191)	0.291
Depth of stromal invasion	0.885 (0.272–2.885)	0.840	2.274 (0.529–9.785)	0.270
Treatment	0.385 (0.106–1.407)	0.149	0.248 (0.045–1.360)	0.108
Respond of neoadjuvant therapy	3.662 (0.754–17.784)	0.107	5.299(0.767–36.612)	0.091

*DFS* disease free survival, *OS* overall survival, *CI* confidence interval, *HR* hazard radio, *NA* not available

## Discussion

Treatment selection for cervical AC should be based on a comprehensive assessment of resource availability. For the purpose of providing evidence-based, resource-stratified global recommendations to policy makers, ASCO takes into account the function of preoperative chemotherapy in areas where resources are limited [19]. Recently, a phase II study showed that dose-dense neoadjuvant paclitaxel/carboplatin is feasible and safe in LACC patients. However, it is unclear how neoadjuvant chemotherapy affects survival [20]. The survival benefit of NACT is currently being evaluated in the EROTC 55994 trial, and thus far, they have found no difference in the 5 year OS between patients receiving NACT + RS and those receiving CCRT [21]. Gupta et al. reported that LACC patients treated with NACT + RS had inferior DFS compared to patients treated with CCRT (*P* = 0.003); the incidences of adverse events in both groups were within acceptable limits. This prospective study showed a rigorous design and high level of evidence, but they included 179 stage IIB patients who underwent RS, and subgroup analysis showed that the difference was mainly due to these patients [22]. Further, this study included only patients with SCC. As there are differences between AC and SCC in epidemiology, biological characteristics, and chemoradiotherapy sensitivity, the best treatment model for cervical AC is likely not reflected in these clinical trials.

In this retrospective analysis, pathological response criteria were used to evaluate the short-term effects of neoadjuvant therapy. These criteria have been used in many multicenter retrospective analyses and balance the shortage of imaging resources. 10 patients in the NACT + RS group achieved PR (41.7%), as did 20 patients in the NACRT + RS group (52.6%). The pathological response criteria are stricter than the Response Evaluation Criteria in Solid Tumors (RECIST), which define PR as residual lesions < 3 mm and no lymph node metastasis. For this reason, PR rates are lower when judged by pathological response criteria. The response rate of neoadjuvant chemotherapy when judged by RECIST criteria fluctuates from 48.4 to 93.0%, and in studies assessing response by pathology, the response rate fluctuates from 27.6 to 30.6% [23, 24]. Several articles also pointed out that pathological response was an indicator for satisfying clinical outcome [16, 25].

Multivariate analysis showed that lymph node metastasis was an independent prognostic factor of survival. The rate of lymph node positivity was 23.1% in our study, and the 5 year survival rate in patients with lymph node metastasis was 37.0%. Baalbergen et al. found that the survival rate in patients with surgically treated stage I–IIB AC was approximately 91% if the lymph nodes were negative, but it dropped to 10–34% if they were positive [26]. Irie et al. found that the incidence of lymph node involvement was significantly higher in patients with AC than in those with SCC (31.6% vs 14.8%) [27]. Mabuchi et al. found that the impact of pelvic nodal metastasis was larger in patients with AC histology than in those with SCC histology (HR: 12.9 versus 3.51), and Cox proportional hazards model indicated a negative response to therapy in AC patients with lymph node metastasis [28].

A number of studies have indicated that AC is not as sensitive as SCC to either radiotherapy or CCRT [10–13]. Yokoi et al. compared the survival outcomes of AC/ASC and SCC patients receiving definitive radiotherapy and found that the 5 year OS rates were 26.7 and 58.6%, respectively, although that study included only 24 AC/ASC patients [7]. One institution in China matched and compared 744 SCC and 71 AC patients who underwent RT or CCRT. They found that patients with AC were more likely to experience recurrence and had worse survival outcomes than patients with SCC [8]. The smaller number of AC patients may be part of the reason for the difference in survival outcomes. Ryu et al. sought to determine a new criterion that included AC as an intermediate-risk factor for recurrence and found that it was more sensitive and specific when compared to the Classic model or the GOG model (*P* = 0.0048) [29]. Further, the survival rate of some subtypes of AC may be worse, such as endometrial endometrioid adenocarcinoma, gastric type adenocarcinoma, or clear cell adenocarcinoma. The biological behavior of cervical AC must also be taken into account. Several studies have investigated survival outcomes in cervical AC to determine more appropriate treatment approaches. One possibility is NACT before CCRT. The use of NACT before CCRT followed by adjuvant therapy in patients with AC histology has been reported. Tang et al. compared NACT before CCRT followed by adjuvant therapy with CCRT alone in 880 patients with stages IIB-IVA AC and observed that sandwich chemotherapy is more effective and safe [30].

Patients in the NACRT + RS group who achieved pOR with neoadjuvant treatment still had poor prognosis (Fig. 3c, d). However, in several retrospective studies, neoadjuvant brachytherapy and chemotherapy followed by RS for stage IB2 and IIA cervical cancer patients had no obvious inferiority to NACT + RS [31, 32]. Additionally, Vízkeleti et al. observed that the postoperative response rate in patients who received preoperative intrauterine brachytherapy was higher than that in patients who received RS alone (*P* = 0.03), and the rate of positive surgical margins was significantly lower (*P* = 0.02) [33]. This suggests that the underlying reason for our results is that radiotherapy and neoadjuvant brachytherapy reduce the pathological response in cervical AC.

This study has some limitations. We only included a small number of patients, and the retrospective analysis is not sufficiently rigorous to settle the question of the effects of neoadjuvant therapy in IB2/IIA2 cervical cancer. Studies with larger sample sizes and multicenter prospective randomized studies are needed to further determine the role of preoperative adjuvant therapy.

In conclusion, there was no significant difference in survival between cervical AC patients who received NACT + RS and those who received RS alone. Patients who responded to NACT had favorable prognosis, which may suggest that there are subgroups in which neoadjuvant therapy may be beneficial. No survival benefit was observed in the NACRT group, even in responders.
